# Optimizing remote heart failure management: First experiences with the HeartInsight score for implanted defibrillators

**DOI:** 10.1002/joa3.13032

**Published:** 2024-03-29

**Authors:** Luca Santini, Francesco Adamo, Nicola Danisi, Karim Mahfouz, Carlo Colaiaco, Ilaria Finamora, Claudia Sorrentino, Mariagrazia Romano, Alessio Ferrara, Paola Napoli, Daniele Giacopelli, Fabrizio Ammirati

**Affiliations:** ^1^ Department of Cardiology Giovan Battista Grassi Hospital Rome Italy; ^2^ Clinical Unit Biotronik Italia Cologno Monzese Italy

**Keywords:** cardiac implantable electronic device, heart failure, HeartInsight, implantable cardioverter‐defibrillator, remote monitoring

## Abstract

We explored the results of two tests of the novel HeartInsight algorithm for heart failure (HF) prediction, reconstructing trends from historical cases. Results suggest potential extension of HeartInsight to implantable cardioverter defibrillators patients without history of HF and illustrate the importance of the baseline clinical profile in enhancing algorithm specificity.

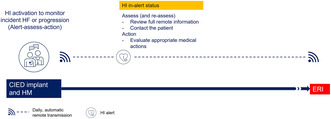

Cardiac implantable devices monitor various parameters associated with patient's clinical status.^1^ Recently, remote monitoring (RM) systems have been improved by incorporating algorithms that combine information from several sensors into a single heart failure (HF) score.^2^ The HeartInsight algorithm (BIOTRONIK SE&Co. KG, Germany) has recently been proposed in patients with implantable cardioverter defibrillators (ICDs), history of HF, and reduced ejection fraction without long‐lasting/permanent atrial fibrillation (AF), showing promising accuracy in predicting HF hospitalizations.^3^ The algorithm combines the temporal trends of seven parameters (24‐h mean and night heart rate, heart rate variability, intrathoracic impedance, AF burden, patient activity, and premature ventricular contractions) with an optional clinical risk stratifier (Seattle HF Model [SHFM]). When the daily HF score exceeds a programmable nominal threshold, HeartInsight triggers an alert indicating an increased risk of worsening HF. No previous case reports have examined the potential application of HeartInsight in clinical practice. In this rapid communication, we retrospectively reconstructed its trends using raw RM data from one case of worsening HF in a patient without history of HF, and from another case of bacterial‐based pneumonia and no cardiac decompensation.

A 67‐year‐old man with coronary artery disease, right ventricle arrhythmogenic dysplasia, first‐degree atrio‐ventricular (AV) block, and no history of HF underwent dual‐chamber ICD implantation (Lumax 740, BIOTRONIK) in 2013 for secondary prevention of sudden cardiac death. The device's pacing mode was set in DDD with a lower rate of 60 beats‐per‐minute and an AV delay of 250 ms to promote spontaneous rhythm. Starting from September 2021, the patient experienced dyspnea with signs of HF, requiring therapy adjustments. Also, new‐onset episodes of AF were observed. For this period, we retrospectively collected medical actions taken, and raw RM data to reconstruct HeartInsight trend (Figure 1). Reconstruction showed an HF alert on August 25, 2021, as the score exceeded the 45 nominal threshold for three consecutive days. The alert would have occurred 12 days before the onset of AF episodes. As reported in our medical records, diuretics were prescribed during an in‐person visit in the subsequent days to manage HF symptoms, which were further confirmed by an elevated B‐type natriuretic peptide level (980 pg/mL). A second HF alert is shown on January 1, 2022 (Figure 1), the same day of an AF recurrence. On January 25th, while still in an in‐alert state, the patient experienced an AF episode of more than 24‐h duration, requiring another outpatient clinic visit where signs and symptoms of worsening HF prompted adjustments to the drug therapy, including initiation of sotalol and an increase in diuretic dosage.

**FIGURE 1:**
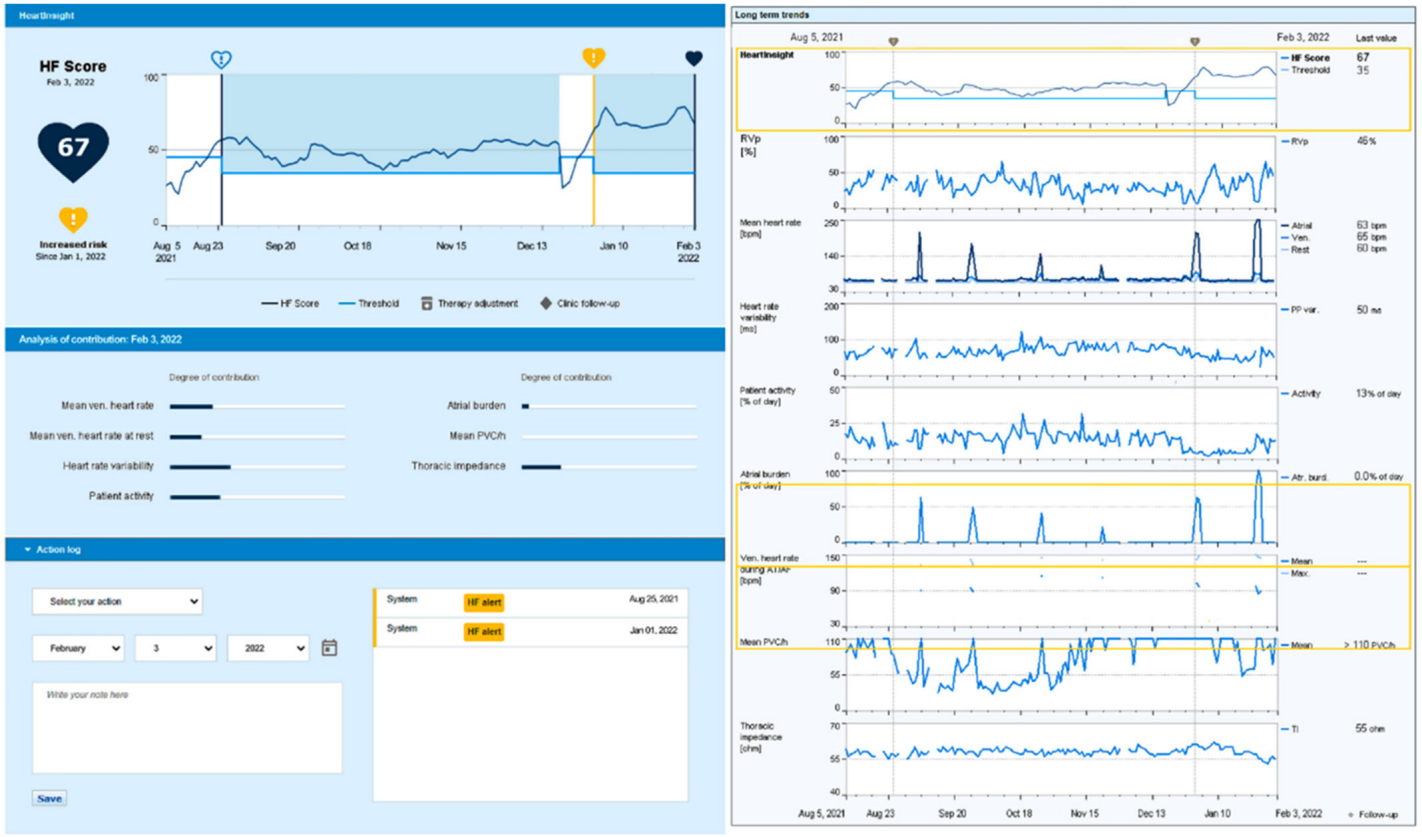
Reconstructed dashboard of the HeartInsight algorithm for Case 1 is presented. The left panel exhibits the current HF score, alert date, the trend of the HF score with the HeartInsight threshold, and an analysis of the parameters contributing to the current score. The right panel displays the trends of all available heart failure diagnostics, which include the percentage of right ventricle pacing, mean heart rates, heart rate variability, patient activity, atrial arrhythmia burden, ventricular heart rate during atrial arrhythmia, the number of premature ventricular contractions per hour and thoracic impedance. On the right, the latest 24‐h value for each variable is provided (dated February 3, 2022). AF, atrial fibrillation; AT, atrial tachycardia; BiV, biventricular; bpm, beats/minute; CRT, cardiac resynchronization therapy; HF, heart failure; PVC, premature ventricular contractions.

A 67‐year‐old man with ischemic cardiomyopathy and reduced left ventricle ejection fraction received an ICD (Intica 5 VR‐T DX, BIOTRONIK) for primary prevention in 2018. At implant, the SHFM score was −0.052. During routine RM surveillance, in fall 2019, RM trends showed a progressive increase in mean heart rate, a decrease in heart rate variability, patient activity, and thoracic impedance, suggesting worsening HF. However, at a phone contact, the patient reported an ongoing pneumonia caused by bacterial infection for which antibiotic treatment had been started a few days earlier (November 23rd). The patient quickly recovered, and on February 4th, during a regular follow‐up, he was found in good conditions. The trend of the HF score was reconstructed (Figure 2) including the SHFM score. The latter resulted in an average reduction of approximately 5% in the value of the score. There was no in‐alert state during the pneumonia, despite individual RM parameters that could have been interpreted as a sign of worsening HF. The SHFM contributed to keep the HeartInsight score below the nominal threshold and prevent a false HF alert.

**FIGURE 2:**
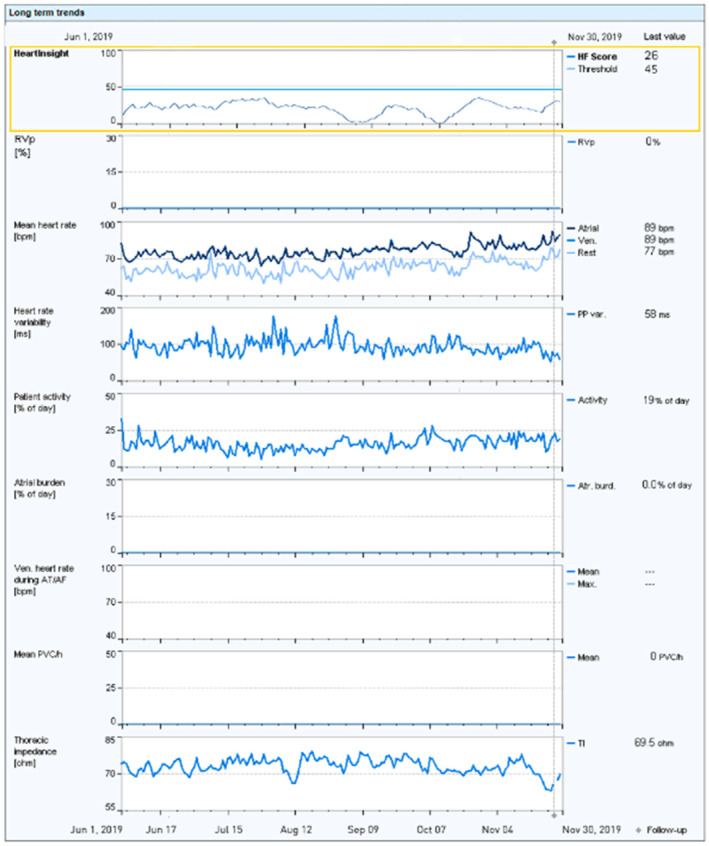
Upper panel illustrates the reconstructed HF score trend, while the lower panel presents all heart failure diagnostics for Case 2, pinpointing the time of bacterial pneumonia diagnosis on November 23, 2019. The patient quickly recovered, and on February 4th, he was found in good conditions. AF, atrial fibrillation; AT, atrial tachycardia; BiV, biventricular; bpm, beats/minute; CRT, cardiac resynchronization therapy; HF, heart failure; PVC, premature ventricular contractions.

We report about two cases of retrospective application of the HeartInsight predictor: In the first case, an alert would have been appropriately triggered before a worsening HF event in a patient with no history of HF; in the second case, the use of SHFM allowed correct classification of a bacterial pneumonia event despite pattern of relevant parameters were suggestive of cardiac decompensation. In the SELENE HF study,^3^ approximately 85% HF hospitalizations presented with normal sinus rhythm. However, in patients with AF, a significant correlation between AF episodes and HF is expected.^4^ In our first case, we observed a connection between the in‐alert state with the occurrence of AF episodes. Interestingly, HF alerts were triggered before the AF onset, as well as before manifestation of signs/symptoms of decompensation that were treated with drug adjustments (initiation or uptitration of diuretics and sotalol).

The second case illustrates the role of the SHFM in specificity enhancement. The negative SHFM score enabled the HeartInsight algorithm to distinguish an ongoing pneumonia from cardiac decompensation despite trends of parameters could have been interpreted as consistent with a worsening HF scenario. The constant negative value of the SHFM score shifted the trend to lower values, thus avoiding a false‐positive HF alert. This mechanism contributed to improve algorithm specificity as observed in the SELENE HF study,^3^ where the optional use of the SHFM resulted in a about 10% reduction in the false‐positive alert rate. The burden of false‐positive alerts can significantly affect the effective remote management of these patients (Figure 3).^5^ Low false‐alert rates are mandatory to increases alert actionability and reduce workload of hospital staff.

**FIGURE 3:**
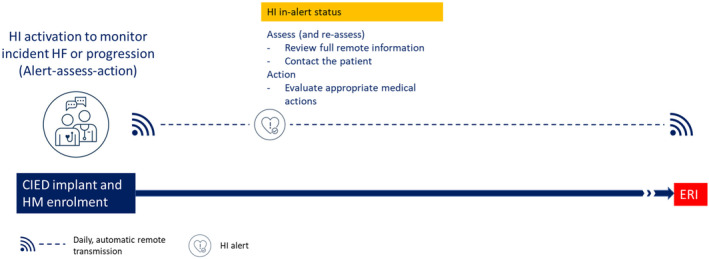
Illustrative example of a time line for patients with cardiac implantable electronic device on remote monitoring and HeartInsight algorithm activation. Alert‐assess‐action is the recommended approach for alert response.^5^ CIED, cardiac implantable electronic device; ERI, elective replacement indicator; HF, heart failure; HI, HeartInsight; HM, home monitoring.

We reported the results of two tests of the novel HeartInsight algorithm generated by reconstructing trends from historical cases. Results suggest potential extension of HeartInsight to ICD patients without history of HF and illustrate the importance of the baseline SHFM in enhancing algorithm specificity.

## CONFLICT OF INTEREST STATEMENT

Luca Santini served as consultant to Abbott, Medtronic, Boston Scientific, and Dompè, and received speaking honoraria for Medtronic, Boston Scientific, Abbott, Microport, and Boeheringher. Alessio Ferrara, Paola Napoli, and Daniele Giacopelli are employees of Biotronik Italia S.p.a. The remaining authors have nothing to disclose.

## PATIENT CONSENT STATEMENT

Patients gave consent to publish this material.

## Data Availability

The data that support the findings of this study are available from the corresponding author upon reasonable request.
